# Dynamical Pattern Representation of Cardiovascular Couplings Evoked by Head-up Tilt Test

**DOI:** 10.3390/e20040235

**Published:** 2018-03-28

**Authors:** Danuta Makowiec, Dorota Wejer, Beata Graff, Zbigniew R. Struzik

**Affiliations:** 1 Institute of Theoretical Physics and Astrophysics, Faculty of Mathematics, Physics and Informatics, University of Gdańsk, Wita Stwosza 57, 80-308 Gdańsk, Poland; 2 Institute of Experimental Physics, Faculty of Mathematics, Physics and Informatics, University of Gdańsk, Wita Stwosza 57, 80-308 Gdańsk, Poland; 3 Department of Hypertension and Diabetology, Medical University of Gdańsk, M. Skłodowskiej-Curie 3a, 80-210 Gdańsk, Poland; 4 Graduate School of Education, University of Tokyo, 7-3-1 Hongo, Bunkyo-ku, Tokyo 113-0033, Japan; 5 RIKEN, Brain Science Institute, 2-1 Hirosawa, Wako-shi 351-0198, Japan

**Keywords:** time series analysis, dynamic symbolization, pattern representation, information transfer, orthostatic stimulus

## Abstract

Shannon entropy (ShE) is a recognised tool for the quantization of the temporal organization of time series. Transfer entropy (TE) provides insight into the dependence between coupled systems. Here, signals are analysed that were produced by the cardiovascular system when a healthy human underwent a provocation test using the head-up tilt (HUT) protocol. The information provided by ShE and TE is evaluated from two aspects: that of the algorithmic stability and that of the recognised physiology of the cardiovascular response to the HUT test. To address both of these aspects, two types of symbolization of three-element subsequent values of a signal are considered: one, well established in heart rate research, referring to the variability in a signal, and a novel one, revealing primarily the dynamical trends. The interpretation of ShE shows a strong dependence on the method that was used in signal pre-processing. In particular, results obtained from normalized signals turn out to be less conclusive than results obtained from non-normalized signals. Systematic investigations based on surrogate data tests are employed to discriminate between genuine properties—in particular inter-system coupling—and random, incidental fluctuations. These properties appear to determine the occurrence of a high percentage of zero values of TE, which strongly limits the reliability of the couplings measured. Nevertheless, supported by statistical corroboration, we identify distinct timings when: (i) evoking cardiac impact on the vascular system, and (ii) evoking vascular impact on the cardiac system, within both the principal sub-systems of the baroreflex loop.

## 1. Introduction

Systems controlling the human body produce complex signals that are usually not stationary and have properties specific to the individual subject from whom the recordings are obtained. Such features hamper the use of many methods aimed at revealing physiological phenomena underlying the dynamics of the system. This also applies to the two major cardiovascular signals: the time interval between subsequent heartbeats (the so-called RR-intervals) and the change in systolic blood pressure values (SBP), which are commonly used in the evaluation of the functional state of cardiovascular control maintained by the autonomic nervous system [1,2]. The autonomic nervous system is considered to be the crucial external regulator assuring cardiovascular homeostasis [3]. The head-up tilt (HUT) test is a procedure that elicits so-called orthostatic stress in a subject by moving his/her body from a relaxed supine position to an upright position. The change in body position results in a transition in neural activity from vagal predominance at rest into strong activation of the sympathetic system while standing [4,5,6].

The activation of the complex network of interactions between the neural and non-neural systems maintaining the proper blood distribution during the HUT test affects the variability of both RR-intervals and SBP values. This rapid transition of bodily orientation is captured in the low- and high-frequency analysis (LF and HF) of RR-intervals: from small LF and large HF in a relaxed supine position to large LF and small HF after moving to an upright position in the HUT test [4]. Similar effects have been observed in the heart rate variability (HRV) indices that quantify rapid changes in the RR-intervals: RMSSD (square root of the mean squared differences between RR-intervals) or pNN50 (percentage of all adjacent RR-intervals that differ by more than 50 ms), which, consequently, have been considered to reveal vagal activity [1,7]. These linear techniques of frequency and time domain analysis have been complemented by many nonlinear techniques concentrating on exposing short-term complex relationships within RR-intervals. One such method presumes studying three, rather than two subsequent RR-intervals and then evaluating their role in the overall signal variability (further referred to as *deterministic patterns*) [8]. Different variants of this approach have been systematically investigated in the quantification of autonomic regulation effects during the HUT test [9,10]. Recently, an alternative classification of three-element segments has been proposed (further referred to as *dynamical patterns*) [11]. This new approach refers primarily to the dynamics of changes, i.e., it aims at capturing whether a three-element segment of a signal represents a speeding up or slowing down of changes in a signal.

There have been observations suggesting that HRV measures are strongly correlated directly with heart rate [12], namely that the standard deviation of RR-intervals (SDNN) decays exponentially with the heart rate [13]. Thus, discussion of the continuous effects of patient-to-patient variety is based on conclusions drawn from HRV [14]. It is considered that, by normalization of signal values, the effect of patient-to-patient variability can be reduced. Therefore, in the following, we pay special attention to the problem of how the data is pre-processed.

Entropic measures, such as Shannon entropy (ShE) and transfer entropy (TE), have been found to be useful for the quantization of the temporal organization of time series [15,16] and for providing insights into dependences between coupled systems; see [17,18]. In particular, these measures can be performed using surrogate data tests, which make it possible to discriminate genuine coupling phenomena from random, incidental fluctuations. Moreover, in the case of real-world signals with unknown underlying dynamical organization, they provide an evaluation of the quality of the symbolization used—this is due to the Minimal Entropy Principle—the smaller the entropy, the better the symbolization [19].

In the following, RR-intervals and SBP signals, firstly min-max normalized and then binned into six discrete values, are symbolized, either using the deterministic or the dynamical pattern coding approach. Properties of the resultant coded signals are then investigated using ShE and TE, in order to obtain information about the physiological transition phenomenon in the cardiovascular system provoked by the HUT test. Namely, we search for the specificity (uniqueness) of the dynamics of RR-intervals and SBP values when the subject is in the resting supine position and subsequently their body is moved into the upright position. Moreover, we investigate how these dynamics differ from purely stochastic dynamics, and how the symbolization used influences the results. Our findings are compared with our earlier estimates obtained using a fixed binning approach [11]. In particular, we discuss why the two approaches: adaptive, patient-oriented binning and fixed, segment-oriented binning, lead to similar conclusions in the case of RR intervals, but, in the case of SBP values, these two approaches do not provide consistent results.

Moreover, in keeping with the baroreflex model of cardiovascular regulation (see, e.g., [20,21,22]), we expect that autonomic control should cause the occurrence of similar sequences of consecutive events in the distinct signals considered. Namely, a growth in SBP values should be accompanied by a growth in RR-intervals and vice versa. Such sequences of growths or falls should be particularly well captured by the dynamical patterns. Therefore, we test how the two different symbolizations applied in signal pre-processing influence the TE estimates of the couplings between the cardiac and the vascular systems. Moreover, we ask whether both these estimates are coherent or not in their description of the couplings obtained and we attempt to identify the physiological aspects of the phenomena.

### Outline of the Paper

Since the objectives of this work are multiple, we provide a detailed outline below.

In Section 2, all of the methods used are introduced and their utility is discussed. After presenting the protocol of the HUT test performed and the group of subjects from whom the signals were recorded (Section 2.1), we subsequently introduce the numerical methods that are applied to these signals. We thoroughly define two methods of pre-processing the signal values and discuss consequences of applying each of these pre-processing procedures (Section 2.3). Then, we explain the two methods of pattern symbolization applied to signal representations (Section 2.4). We also briefly describe the surrogate tests that we perform in justifying the reliability of the estimates (Section 2.5). The concept of TE is explained in the Section 2.6.

Section 3 contains our main results together with a discussion of these results. The results are presented in three parts corresponding to the leading problem considered. The first part (Section 3.1) refers to distributions of patterns resulting from the applied signal pre-processing methodology. These distributions are the reference point for all further entropy estimates. Then, in Section 3.2, we present the estimates of ShE. Our discussion concentrates on the insight provided by signals mapped into patterns in the context of the physiology of the HUT test. In particular, we explain how the signal value pre-processing applied could lead to deceptive results. The third part of Section 3 is devoted to the investigation suggested in the title of the manuscript, namely to the possibility of revealing the differences in dynamic organization of physiological processes evoked by the HUT test. In particular, we focus on the dynamical pattern representation, which is employed here to demonstrate whether and how the relationships anticipated within the baroreflex regulatory loops are captured and revealed using the TE.

The last section, Section 4, summarizes and concludes our results.

## 2. Materials and Methods

### 2.1. Signal Acquisition

The study group consisted of fifty healthy humans who had not experienced syncope in their life. The experimental protocol complied with the Declaration of Helsinki and was approved by the Bioethics Committee of the Medical University of Gdańsk. All of the subjects gave their informed written consent to participating in the study.

The subjects were placed on a tilt table with a footboard support. After attaching the electrodes for electrocardiography (ECG) and blood pressure cuffs, the subjects relaxed for 30 min in a supine position in a comfortable environment. The arm with the adjusted finger cuffs was positioned so that the lower arm remained near the hydrostatic indifference point in any posture. Then, the table was tilted to 60°. The study participants remained in the upright position for 20 min or until the occurrence of syncope. If fainting did not occur, the subjects were administered 400 micrograms of nitroglycerine (aerosol, sublingually) and the test was continued for 10 min or until syncope occurred. The test was performed under a paced breathing protocol—during the whole test, the subjects were asked to synchronize their breathing rhythm with recorded voice instructions to breathe in and out at a frequency of 0.25 Hz.

The results of the HUT test were interpreted according to the modified Vasovagal Syncope International Study (VASIS) classification [23]. If blood pressure fell, followed by an increase in RR-intervals to less than 1.5 s, or if the RR-intervals increased to more than 1.5 s, and this increase lasted less than 10 s (so asystole did not occur), the syncope is said to be of mixed type. If the RR-intervals increased to an asystole of more than 3 s, or RR-intervals of a size greater than 1.5 s lasted longer than 10 s, together with or before a drop in blood pressure, the syncope is described as cardioinhibitory.

Fourteen of the subjects fainted after administration of nitroglycerine in a mixed type syncope condition. Their signals were excluded from the study group. Nine patients were eliminated from the study because of the poor quality of their recordings. The group studied, denoted as the control group (CG), ultimately consisted of twenty-seven patients.

### 2.2. Data Pre-Processing

**Figure 1:**
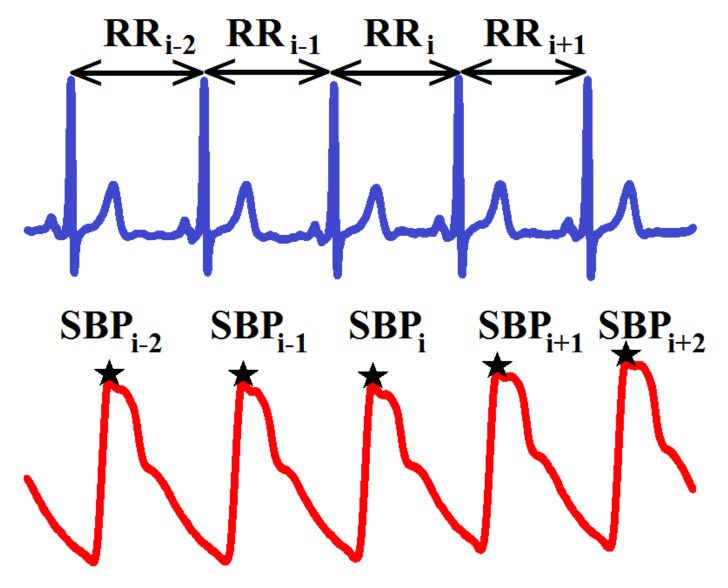
An extract from a typical recording of ECG (**top**) and continuous blood pressure (**bottom**) with references to series investigated with RR-intervals $RR_{i}$ and systolic blood pressure $SBP_{i}$.

The simultaneous recordings of ECG and continuous blood pressure were obtained noninvasively and automatically evaluated using a computer-based system (Task Force Monitor, CNSystems, Graz, Austria); see Figure 1. This continuous blood pressure measurement is considered to be effectively equivalent to that of Finapres (Ohmeda, Louisville, CO, USA) [24]. The ECG and beat-to-beat blood pressure signals were sampled at a frequency of 1000 Hz, and changes in thoracic impedance due to respiration at 50 Hz. All RR-intervals are represented in are represented in milliseconds [ms] and SBP levels in millimeters of mercury [mmHg]; see plots in Figure 3A,E.

**Figure 2:**
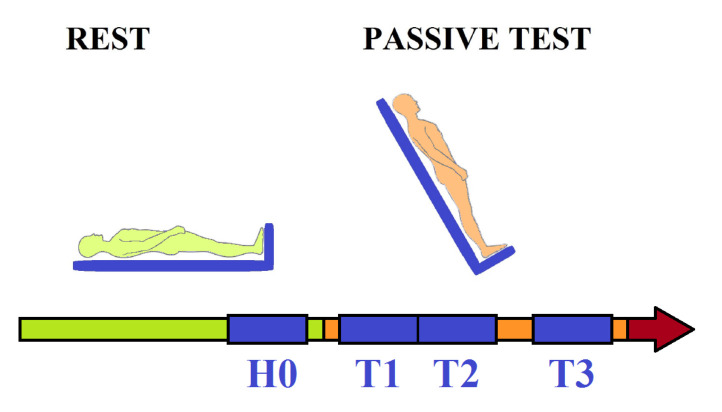
Basic scheme of the head-up tilt (HUT) test. The first part—rest in a supine position, which switches into the so-called passive part after tilting, during which syncope is expected to develop by itself. Then the active part starts (marked by the red arrow) with administration of nitroglycerine, which is supposed to advance the occurrence of syncope. Signals are observed in the four time windows: H0, T1, T2 and T3. Each window represents 300 successive points.

The cardiovascular response to orthostatic stress was observed in four time windows: a time period before the tilt H0 and three periods after the tilting: T1, T2, T3; see Figure 2. In order to eliminate the influence of instabilities occurring in a signal when tilting, the period H0 ended one minute before tilting. For the same reason, T1 started one minute after tilting. The period T2 started just after T1, while T3 ended 1 min before the end of the passive part of the test. Each time window contains 300 consecutive signal points.

### 2.3. Data Symbolization

#### 2.3.1. Min-Max Signal Normalization and Equal-Interval Binning

Let $X=\{x_{i},i=1,\ldots ,N\}$ denote a sequence with either RR-intervals or SBP values. To normalize each signal value to a [0,1] interval, the following min-max transformation was applied

(1) $$x_{i}\to \frac{x_{i}-\min\left(X\right)}{\max(X)-\min(X)},\;i=1,\ldots ,N.$$

**Figure 3:**
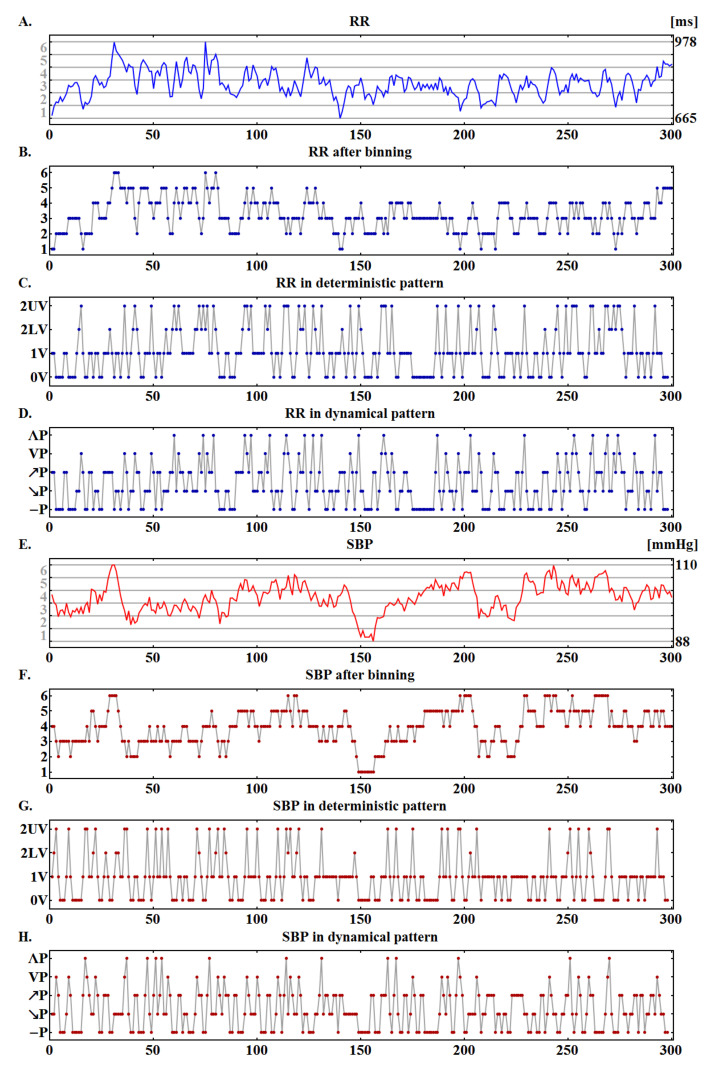
Illustration of a six-value signal representation. Original signals with RR-intervals (**A**) and systolic blood pressure values (SBP) (**E**), their six-valued representations (**B**,**F**), and pattern signals: deterministic (**C**,**G**) and dynamical (**D**,**H**), respectively.

Then, each normalized value $x_{i}$ was discretized using uniform binning. We used six uniformly spread sub-intervals over [0,1] as bins. Consequently, each sequence became a signal operating on six values only, which can be represented as ordinal numbers 1, …, 6. In Figure 3, we show how the operations described influenced typical series with RR-intervals and SBP values.

By definition, the approach of min-max normalization applied, together with binning into the fixed number of bins, provides a different bin size for each individual signal. Therefore, such an adaptive binning approach can be seen as patient-oriented binning. However, the size of such an adaptive bin will be highly sensitive to artefacts and, in general, to extreme values. Such extreme values can appear incidentally in artefact-free recordings, especially in signals of young humans, known for their high HRV. Therefore, in practice, one extracts the part of the recording that exhibits the smallest variation (see e.g., [25,26]). However, when a study includes subsequent time windows and envelops the entire recording duration, there is no such freedom. Therefore, to limit the influence of extreme singular values in our studies, we replaced the signal maximum and minimum values with the value next to the minimal or maximal value, respectively.

#### 2.3.2. Pattern Representation of Signal Values

It is straightforward to verify that, for a sequence of any three values $\left(x_{1},x_{2},x_{3}\right)$, there are thirteen possible order relationships. These relationships can be indexed by numbers of three digits, which symbolize the order among the elements as follows:

(111) iff $x_{1}=x_{2}=x_{3}$
(112) iff $x_{1}=x_{2}<x_{3}$
(121) iff $x_{1}=x_{3}<x_{2}$
(122) iff $x_{1}<x_{2}=x_{3}$
(211) iff $x_{1}>x_{2}=x_{3}$
(212) iff $x_{2}<x_{1}=x_{3}$
(221) iff $x_{1}=x_{2}>x_{3}$
(123) iff $x_{1}<x_{2}<x_{3}$
(132) iff $x_{1}<x_{3}<x_{2}$
(213) iff $x_{2}<x_{1}<x_{3}$
(231) iff $x_{3}<x_{1}<x_{2}$
(312) iff $x_{2}<x_{3}<x_{1}$
(321) iff $x_{3}<x_{2}<x_{1}.$

The enumerated representation of a sequence of three-digit values, here referred to as an *ordinal pattern* representation, is ‘de facto’ a revisited version of the permutation pattern representation proposed by Bandt and Pompe [27], which considers weak inequality relations only. Furthermore, the ordinal pattern representation can easily be generalized to segments consisting of any number of values [28,29].

**Figure 4:**
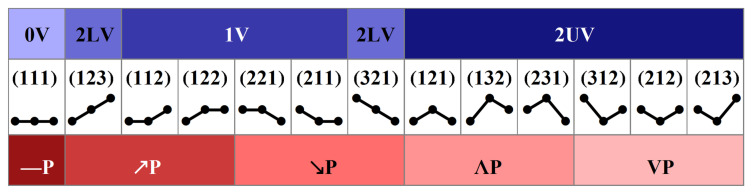
Relations between deterministic and dynamical patterns as classes of three-element ordinal patterns.

We apply the ordinal pattern symbolization to any three-element segments while moving along the pre-processed signal values. We then group patterns into classes that represent specific signal properties (see Figure 4), such as:

*deterministic*: stability versus variations [8]
0V ‘no variation’ pattern: (111);
1V ‘one variation’ patterns : (112), (122), (221), (211);
2LV ‘two like variation’ patterns: (123), (321);
2UV ‘two unlike variation’ patterns: (121), (132), (231), (312), (212), (213).
*dynamical*: growth versus fall [11]
—P ‘flat’ pattern: (111)
↗P ‘growth’ patterns: (123), (112), (122);
↘P ‘fall’ patterns: (321), (221), (211);
ΛP ‘cap’ patterns: (121), (132), (231);
VP ‘cup’ patterns: (312), (213), (212).

#### 2.3.3. Segment Equal-Interval Binning and Pattern Representation

For a given signal resolution Δ, one can apply an alternative binning approach, so-called segment-oriented binning, recently introduced in Wejer et al. [11]. In this approach, the three signal values of a segment $x^{i}=\left(x_{i},x_{i+1},x_{i+2}\right)$ are mapped into a new representation of the segment values $\phi^{i}=\left(\phi_{i},\phi_{i+1},\phi_{i+2}\right)$ by the formula

(2) $$\phi_{i+j}=\left\lfloor \frac{x_{i+j}-\min\left\{x_{i},x_{i+1},x_{i+2}\right\}}{\Delta }\right\rfloor ,\;j=0,1,2,$$

where $\left\lfloor \ldots \right\rfloor$ denotes the largest integral number not exceeding the estimated value. In consequence, in each segment, the bin levels for the signal value symbolization follow the minimal signal value of the segment considered. Then, the vector of symbols $\phi^{i}$ obtained serves for determination of the pattern of the segment.

**Figure 5:**
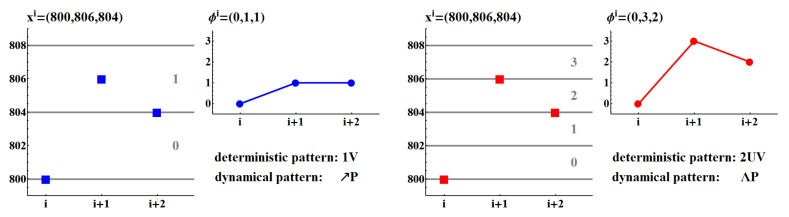
Patterns resulting from a sequence (800, 806, 804) when the method of segment-oriented symbolization is applied with two resolutions: Δ=4 (**left**) and Δ=2 (**right**).

In order to provide more detailed insight into this method, let us consider a segment with values (800, 806, 804); see Figure 5. In the case of Δ=4, Equation (2) leads to $\phi^{i}=\left(0,1,1\right)$, but in the case of Δ=2, Equation (2) shows $\phi^{i}=\left(0,3,2\right)$. Accordingly, the following patterns are assigned when Δ changes: deterministic: 1V → 2UV, and dynamical: ↗P→ΛP. Hence, the small values of Δ lead to more frequent detection of variations in a signal, while for large Δ many of these variations disappear.

### 2.4. Shannon Entropy (ShE)

Let us denote as $S_{\det}=$ { 0V, 1V, 2LV, 2UV } the state space of deterministic patterns, as $S_{\mathrm{dyn}}=$ {—P,↗P, ↘P, ΛP, VP } the state space of dynamical patterns, and as S any space from the above two. Then, a signal $X^{p}=\left(x_{1}^{p},\ldots x_{N-2}^{p}\right)$ where $x_{i}^{p}=\left(x_{i}^{p},x_{i+1}^{p},x_{i+2}^{p}\right)$, $x_{i}^{p}\in S$ is a pattern representation of a signal *X*; see Figure 3. Let p(k) denote the probability of encountering a pattern k∈S in a sequence $X^{p}$. ShE is defined as:

(3) $$H\left(X\right)=-\sum_{k\in S}p\left(k\right)\ln p\left(k\right).$$

ShE provides an evaluation of how the actual distribution of patterns differs from the uniform distribution, where each pattern has the same probability of occurring [30]. In particular, Equation (3) delivers 0 if all the values in a signal take the same value, and the maximal value is achieved for the uniform distribution. It equals ln of the number of all the patterns considered. Hence, it is ln4≈1.39 in the case of the deterministic representation and ln5≈1.61 in the case of the dynamical representation.

### 2.5. Tests with Surrogate Signals

It is to be expected that the limit ShE for any distribution, as distinct from the uniform one, is achieved when values in a signal are randomly tossed. In order to find the actual maximal values for a given signal, we performed estimates with the original signals randomly shuffled. For each RR-interval and SBP signal, a hundred shuffled signals were simulated. Then, pre-processing and symbolization steps were applied and *H* for each pattern representation was found.

### 2.6. Transfer Entropy (TE)

TE describes how past values of interacting processes *X* and *Y* influence the prediction of the present value of *X* [17].

Let us denote as $S_{X}=S_{X,i}$ the state space representing the present values of *X*, and $S_{X^{-}}=S_{X,i-1}\times S_{X,i-2}\times \ldots$, and $S_{Y^{-}}=S_{Y,i-1}\times S_{Y,i-2}\times \ldots$ state spaces describing the past $X^{-}$ and $Y^{-}$ of processes *X* and *Y*, respectively, which in general are assumed to be unlimited. Then, TE from *Y* to *X* is defined as follows [17]:

(4) $$TE_{Y\to X}=\sum_{\left(k_{X},k_{X^{-}},k_{Y^{-}}\right)\in S^{*}}p\left(k_{X},k_{X^{-}},k_{Y^{-}}\right)\ln\frac{p(k_{X}|k_{X^{-}},k_{Y^{-}})}{p(k_{X}|k_{X^{-}})},$$

where $k_{X}\in S_{X}$, $k_{X^{-}}\in S_{X^{-}}$, $k_{Y^{-}}\in S_{Y^{-}}$, and $S^{*}=S_{X}\times S_{X^{-}}\times S_{Y^{-}}$. Hence, TE, while quantifying the influence on system *X* of system *Y*, measures the difference between two conditional entropies: $TE_{Y\to X}=H\left(X|X^{-}\right)-H\left(X|X^{-},Y^{-}\right)$, which states that TE is always non-negative and TE equals zero if there is no coupling between signals *X* and *Y*.

In practical application of Equation (4), the length of the state spaces of the past $S_{X^{-}}$, $S_{Y^{-}}$ must be limited. Let us set this limit at *L*. In our considerations here, we assume L=5, but a number of results when the memory vector was elongated to L=10 are also presented. All of our TE estimations were performed using a non-uniform embedding algorithm [31], which reduces the volume of state spaces $S_{X^{-}}$, $S_{Y^{-}}$ to only the most influential delays in each signal.

In accordance with the special relation between RR-intervals and SBP values shown in Figure 1, the state space $S_{{{\mathrm{SBP}}^{p}}^{-}}$ used to find the most influential delays is dependent on the direction of the estimated interactions. Namely, for $TE_{RR^{p}\to SBP^{p}}$, we consider $S_{{{\mathrm{SBP}}^{p}}^{-}}=S_{{\mathrm{SBP}}^{p},i-1}\times S_{{\mathrm{SBP}}^{p},i-2}\times \ldots S_{{\mathrm{SBP}}^{p},i-5}$, but for $TE_{SBP^{p}\to RR^{p}}$, it is $S_{{{\mathrm{SBP}}^{p}}^{-}}=S_{{\mathrm{SBP}}^{p},i},S_{{\mathrm{SBP}}^{p},i-1}\times S_{{\mathrm{SBP}}^{p},i-2}\times \ldots S_{{\mathrm{SBP}}^{p},i-5}$. Independently of the direction of interaction, the state space of past RR-intervals is defined as $S_{{{\mathrm{RR}}^{p}}^{-}}=S_{{\mathrm{RR}}^{p},i-1}\times S_{{\mathrm{RR}}^{p},i-2}\times \ldots S_{{\mathrm{RR}}^{p},i-5}$.

### 2.7. Tests with Surrogate Signals

It is to be expected that, if signals with RR-intervals and SBP values are misaligned in time with respect to each other, we should not observe entropy transfer between them. We tested this expectation by considering a hundred pairs of signals where random shifts in time (uniform distribution for 20 to 280 beats) between RR-intervals and SBP were applied.

### 2.8. Numerical Estimates and Statistical Analysis

All entropy calculations and randomization of signals were done with *MATLAB R2016A* (MathWorks Inc., Natick, MA, USA) using our scripts or, in the case of TE, scripts adapted from [31].

The Shapiro–Wilk (SW) test was used to verify the normality of the pooled data. Differences between the means in the groups were estimated by one-way analysis of variance (ANOVA) with a post hoc Duncan test. In cases when the normality test failed (p<0.05), the Kruskal–Wallis one-way ANOVA on ranks was automatically applied. Since the time groups consisted of the same patients, one-way repeated measures ANOVA (RM ANOVA) were also performed. A paired *t*-test (or Wilcoxon signed rank test when appropriate) was utilized to test the difference between individual subjects. An unpaired *t*-test (or Mann–Whitney rank sum test when appropriate) was carried out to check the difference between parameters derived from different groups. The McNemar test with Yates correction was applied to the paired data for transfer entropy results in order to verify the significance of the proportions of observations. SigmaPlot 13.0 software (Systat Software, Inc., San Jose, CA, USA) was utilized in all the tests.

## 3. Results and Discussion

Since entropy quantifies distributions of events, we start with a discussion of the properties of the distributions of which the patterns considered are events. Next, we present the resulting entropies and discuss their properties. Finally, we describe couplings in the cardiovascular system evoked by the HUT test, which are detected and quantified by TE.

### 3.1. Distributions of Patterns

Patient-oriented binning has been found to be significant in assessing cardiac autonomic control and predicting cardiovascular health [13,14] as it introduces an individual, adaptive resolution of events for each subject. Consequently, each signal, mapped into six values, allows the separation of events with different timescales; however, this is, in a way, specific to the individual. It is, nevertheless, evident that such restricted binning does limit the discernment of certain heart rate accelerations or decelerations and certain blood pressure rises and falls. For example, in the case of the signals shown in Figure 3, changes in RR-intervals smaller than 52 ms and changes in SBP smaller than 3.6 mmHg may become flattened. In the remaining data, the mean bin in the case of RR-intervals spreads from 45±2 ms in T2 to 53±4 ms in H0. For SBP, the mean bin takes values from 3.6±0.2 mmHg in H0 to 4.7±0.3 mmHg in T3. In the case of RR-intervals, one can consider such large binning as appropriate for distinguishing changes caused by vagal activity from changes due to other factors. Accordingly, one can relate the larger binning in H0 to stronger vagal activity presumed to occur in that part of the HUT test in keeping with commonly accepted knowledge of physiology [4,5,6]. The bin size of SBP values may, therefore, be supposed to reveal effective actions of the known mechanisms regulating blood pressure: the sympathetic activity and the mechanisms related to endothelium [22]. Consequently, as the increase in the bin size is observed with the passing time during the HUT test, one can hypothesize that the mechanisms regulating the blood pressure are gradually activated, leading to an acceleration of changes in blood pressure. Our observations are supported by the physiology of blood pressure regulation, where it is known that prolonged sympathetic activity provokes the emergence of mechanisms related to endothelium [22].

**Figure 6:**
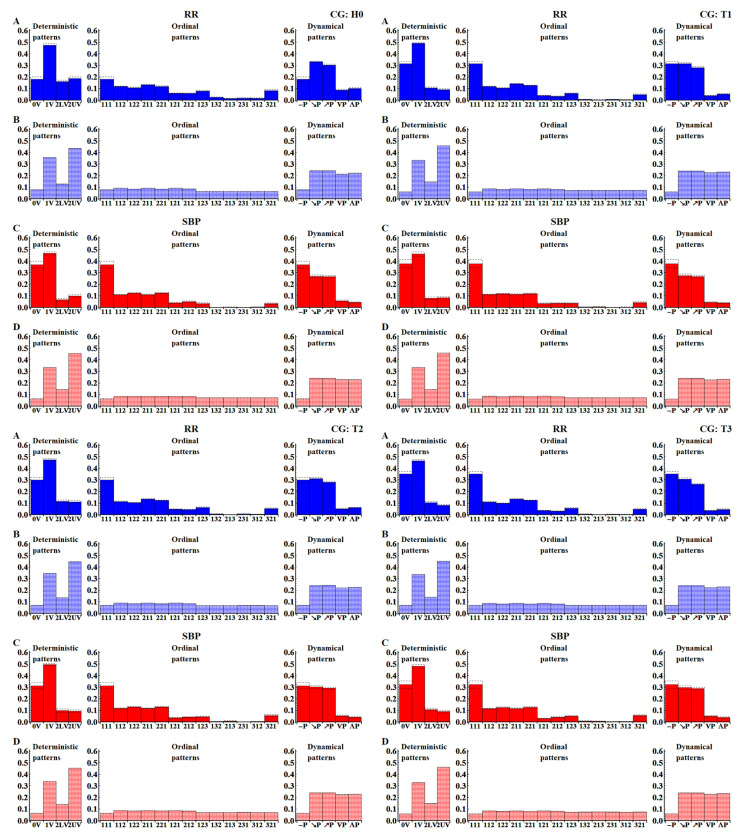
The probability distributions of patterns obtained by averaging over 27 original series with RR-intervals (**A**) and SBP (**C**) represented as sequences of deterministic, ordinal and dynamical patterns in the subsequent time windows. The mean results provided by the corresponding groups with shuffled signals are shown together by the lighter (and checkered) coloured bars in all the panels (**B**,**D**). The dotted lines show standard errors of means.

Figure 6 shows the probability distributions of patterns obtained by pooling the results according to the specific pattern representation: deterministic, dynamical or ordinal, and to the type of signal: original or shuffled values. One can observe that what clearly distinguishes the original signals from the shuffled ones is the role of the flat pattern, i.e., pattern ‘**(111)**’ ≡ ‘0V’ ≡ ‘−P’. In all the time windows, there is more evidence of flat patterns in the unperturbed signals than in the shuffled signals. The transition to the upright body position results in a gradual increase of the probability of observing a flat pattern in the subsequent time windows in the case of the RR-intervals. The low participation of the flat patterns in the RR-signals in the H0 time window remains in agreement with the above-mentioned interpretation, i.e., it results from the domination of the vagal activity in the relaxed supine position. Based on the known physiology of orthostatic stress [4,6,22], the abundance of flat patterns in the T1–T3 time windows should rather be related to slow variability, which emerges after the tilt due the withdrawal of vagal activity and the simultaneous activation of the sympathetic system. The opposite effect, i.e., a gradual decrease in the probability of encountering a flat pattern in subsequent time windows of SBP values, is observed. Instead, one observes a considerable presence of ordinal patterns **(112) (122) (211) (221)**, which may be associated with slow changes. This results in the increased presence of the ‘1V’ deterministic pattern, and ‘↗P’ and ‘↘P’ dynamical patterns. Effectively, signals represented by the deterministic patterns could be seen as two-valued signals, and signals represented by the dynamical patterns as three-valued signals.

It should be stressed that, for the randomly shuffled signals, approximately uniform distributions were observed only in the case of the ordinal pattern representation. Distributions obtained when signals were represented by the dynamical patterns show equal probability for all the patterns except for the flat pattern ‘−P’. This perfectly agrees with the pattern representation presented in Figure 4, as all the patterns except ‘−P’ are represented by the three ordinal patterns, which are equally likely to occur. Deterministic patterns led to evident bi-modal distributions with peaks ‘1V’ and ‘2UV’. This also agrees with the pattern representation presented in Figure 4, as these two patterns are represented by four or six ordinal patterns. Accordingly, it can be suggested that the ordinal patterns are a good signal representation for revealing specific properties of cardiac time series dynamics. However, in the case of short recordings, statistics based on 13-value signals might not be satisfactory, especially when investigations are aimed at evaluating transfer entropy.

### 3.2. ShE of Patterns

Our investigations on ShE lead us to state the hypothesis that after a sharp response occurring immediately after the change in body position in the T1 window, when the entropies in both the symbolizations diminish, the cardiac regulation produces relaxation in the cardiovascular system in the T2 time window. Then again, the system is actively stimulated in T3 to reach the optimal state. Below, we present arguments for this scenario.

#### 3.2.1. Group Means

Shannon entropy is a powerful concept in information theory. If one knows the dynamics underlying the observed signal, usually there is no ambiguity regarding the symbolization procedure [19]. However, in the case of real-world processes, such as for example physiological series, the information content in general depends on the symbolization procedure used. The Minimal Entropy Principle states that, in the case of signals with unknown dynamics, the minimum over all the possible symbolization procedures leads to the most reliable ShE estimate. In Table 1, we present the ShE resulting from the mean group distributions shown in Figure 6. In Table 2, we present the mean entropy calculated by pooling the entropies obtained from the individual patients’ signals, according to the pattern and time window groups. From Table 1 and Table 2, one reads that the deterministic symbolization provides smaller values than the dynamical symbolization. However, after normalization of these values by the state space size, the two symbolizations provide similar values. Hence, both symbolizations—the deterministic and the dynamic—appear to provide an equivalent description of the dynamical changes in the signals considered.

Standard interpretations of ShE values cannot be applied here because the distributions of deterministic and dynamical patterns obtained from the signals with shuffled data are not uniform; see Figure 6. This especially concerns the case when the deterministic pattern symbolization is used. This can be demonstrated by the entropy estimates calculated directly from the distributions presented in Figure 6 and in Table 1. In particular, we see that the shuffled data gives even lower values when the signals are represented by the deterministic patterns. In this case, both the distributions: the distribution obtained from the real signals and the distribution provided by the shuffled signals are bi-modal. The difference between these distributions is that, instead of a 2-variation peak, the original signals peak at zero-variation.

In general, the entropy shown in Table 2 is lower than the corresponding entropy in Table 1. This results directly from the nonlinearity of ShE estimates. However, in both cases, we observe a decrease in ShE in the T1 time window for all the types of representations and for all the types of signals. This decrease could be interpreted as the measure of the first response of the regulatory system to maintain homeostasis after the orthostatic stress.

**Figure 7:**
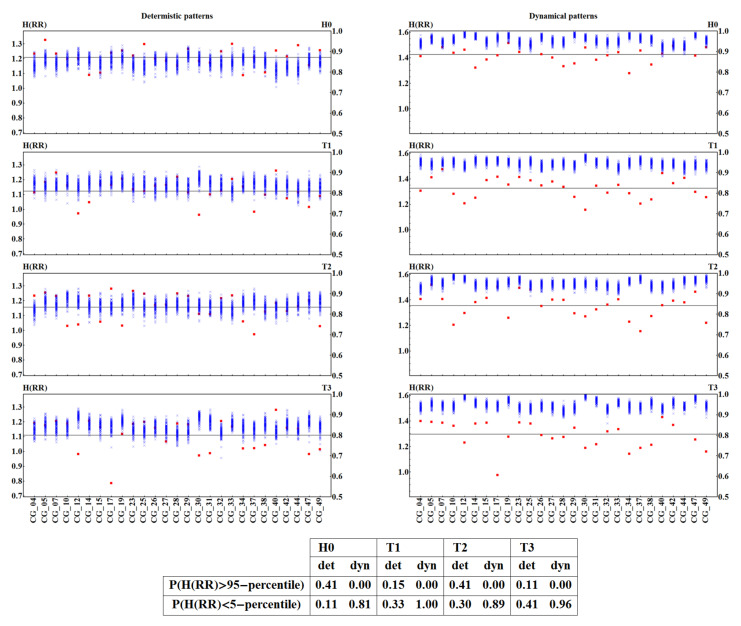
ShE for 27 signals with RR-intervals recorded in each time window H0, T1, T2, and T3 in subjects from the CG group and for their hundred shuffled surrogates. ShE values are divided into results obtained from signals represented by deterministic patterns (**left** column) and dynamical patterns (**right** column). Red squares mark values obtained for original signals. Blue crosses indicate results for surrogates. The right axis shows values of normalized entropy. Grey lines denote the group means. Below the plots, in the table, the probability is given of observing a ShE value different from the incidental one for the two signal representations and in subsequent time windows.

**Figure 8:**
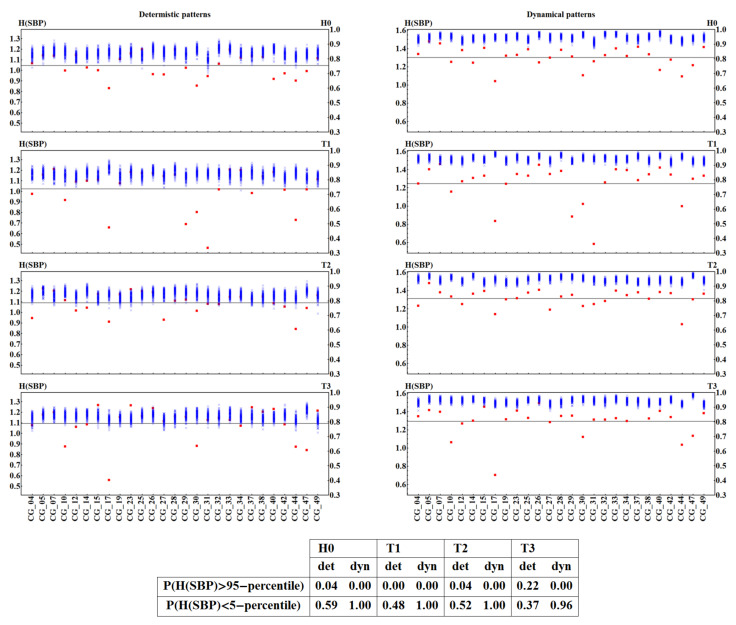
ShE for 27 signals with SBP values recorded in each time window H0, T1, T2, and T3 in subjects from the CG group and for their hundred shuffled surrogates. ShE values are divided into results obtained from signals represented by deterministic patterns (**left** column) and dynamical patterns (**right** column). Red squares mark values obtained for original signals. Blue crosses indicate results for surrogates. The right axis shows values of normalized entropy. Grey lines denote the group means. Below the plots, in the table, the probability is given of observing a ShE value different from an incidental one for the two signal representations and in subsequent time windows.

The pattern representation induces strong two-time-step correlations in a time series representing the original signal. This will also modify the standard ShE interpretation. In order to filter out these correlations, we studied the distance between entropies obtained from the random arrangement of values in a signal and entropy obtained from unperturbed physiological signals. The results are presented in Figure 7 and Figure 8, where together with a patient’s ShE, we plot all the values obtained from shuffled signals. It can be observed that ShE from shuffled signals shows entropy values independent of the patient and the time window, although for the original signals the value of ShE changes from patient to patient and in different stages of the HUT test.

In particular, in Figure 7 and Figure 8, we see a quite evident separation between the signal values and the shuffled data, when the signals are represented by the dynamical patterns. This separation appears to reveal a special organization of signal values when compared to the random arrangement. In the case of a deterministic pattern representation, there are signals that provide entropy indistinguishable from the shuffled data, which can be directly verified from the tables in Figure 7 and Figure 8.

Finally, we have to compare the results described above concerning ShE values with the ShE estimations obtained for pre-processing using common binning for all signals, i.e., with the results that were reported in [11] for the same pool of signals. There, it was found that, under pre-processing with the same bin common to all the signals, if the bin size was large enough, the RR-intervals resulted in a decrease in ShE in the T3 window, while the SBP values resulted in an increase in the T3 window. This observation was found for both types of symbolizations used in Wejer et al. [11]. However, in the present case, when the adaptive, patient-oriented binning is used, the distinction between rest and the tilted position is not as clear as that found when the common binning was applied. This was independent of the pattern representation used. We discuss this observation in the next subsection.

#### 3.2.2. Individual Traces of ShE of Patterns

**Figure 9:**
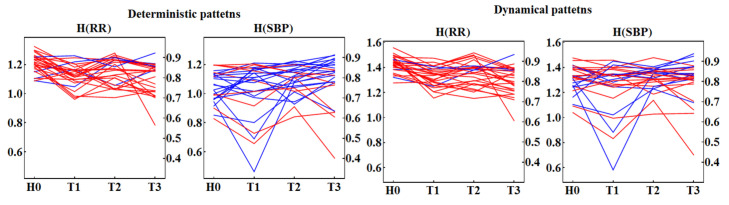
Traces of ShE obtained from 27 signals with RR-intervals and SBP recorded in each time window H0, T1, T2, and T3. The red colour is used for signals for which entropy in H0 is greater than in T3. The blue colour marks signals for which the opposite relation is observed. Friedman one-way repeated measures ANOVA on ranks provides: deterministic patterns—RR-intervals indicate statistically significant differences in the group medians (p<0.001); the pairwise comparisons (Tukey test) of the groups show that H0 is different from both groups T1 and T3. SBP values indicate statistically significant differences in medians among the groups (p=0.04), but the pairwise comparison (Tukey test) does not show any pair to be significantly different; dynamical patterns—RR-intervals exhibit statistically significant differences in medians (p<0.001); the pairwise comparisons (Tukey test) show the H0 group to be different from the groups T1, and T3; SBP does not reach statistical significance for differences in medians (p=0.52).

By grouping subjects according to the time windows of the HUT test, the individual variability of a subject could be lost. To test how signals for a given subject change under the condition of the HUT test, and how the signals vary between individuals, we study alterations in ShE in subsequent time windows of the HUT test for each subject separately. In Figure 9, the entropy of each individual patient is shown, but in a patient-specific follow-up using the adaptive binning procedure. The patients’ signals are divided into two groups—those that are in agreement with the findings of Wejer et al. [11] and others. This approach significantly improves the statistical power of the test for the group discrimination; see notes under Figure 9. In particular, we see that, in both symbolizations for RR-intervals, the entropy in the T2 window appears to be statistically indistinguishable from the entropy obtained in H0. In the remaining time windows: T1 and T3, the entropy is distinct from the entropy in H0. ShE is mostly lower in T1: 22 cases of 27 considered in the deterministic and 23 cases in the dynamical symbolization. In T3, ShE is also mostly lower: 23 cases in the deterministic and 24 cases in the dynamical symbolization. The one-sided Mann–Whitney test for lower entropy in T3 than in H0 in both pattern representations gives high significance (*p* < 0.0001) to the hypothesis that $H_{H_{0}}^{\mathrm{RR}}>H_{T_{0}}^{\mathrm{RR}}$.

However, the hypothesis of greater value of entropy estimated from SBP values in the T3 window when compared to the H0 window is not obvious. For signals represented by the deterministic patterns, we count 18 such cases, which in the one-sided Mann–Whitney test leads to satisfactory statistical significance (p=0.16). For the dynamical pattern representation, the presence of only 11 such cases does not support the hypothesis of the entropy increase in the late tilt time window.

#### 3.2.3. ShE and Different Binning Methods

**Figure 10:**
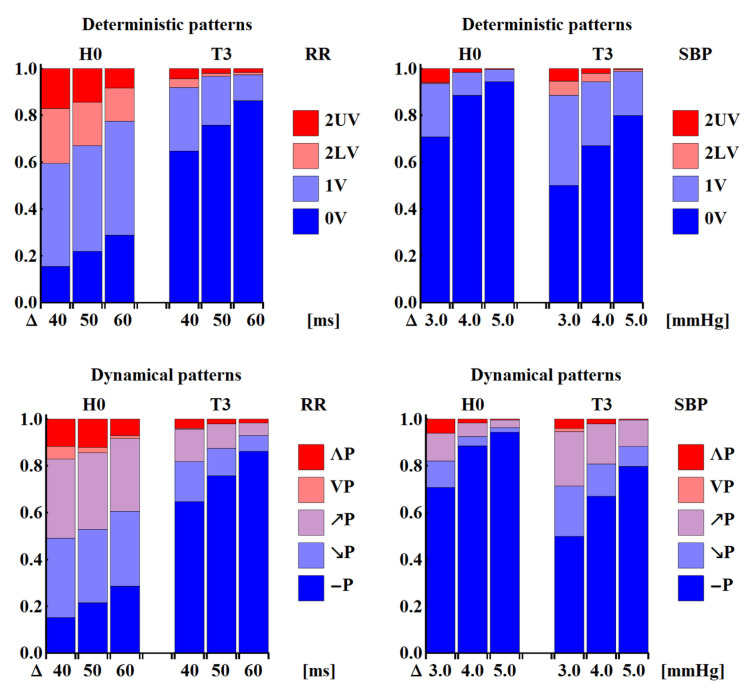
The distributions of patterns observed in signals binned according to segment equal-interval binning (Equation (2)). The sizes of bins (denoted on the horizontal axis) are chosen to correspond with the mean bin observed at a patient’s min-max normalization binning.

Having still no clear answer as to whether the SBP entropy in the late tilt is higher than the SBP entropy in the supine position, we revised the estimates of Wejer et al. [11], namely estimates performed on signals pre-processed with segment equal-intervals binning; see Section 2.3.3. The distributions obtained with bins of sizes comparable to the mean bins resulting from the binning after the patient min-max normalization (Equation (1)) are shown in Figure 10. The variability of events based on RR-intervals in H0 is distinguishably larger than in T3, while, in the case of SBP, the opposite relation holds, which is made evident in Figure 10. This clearly explains the reasons for the observed decrease in the pattern entropy for RR-intervals and the increase of the pattern entropy for SBP values. Furthermore, Figure 10 clearly illustrates why, in the case of RR-intervals, this decrease survives mixing of bin sizes—the effect of binning after the min-max normalization of each signal. However, it is not true in the case of SBP. Here, the distributions corresponding to bin = 3 mmHg in H0 and bin = 5 mmHg in T3 are similar to each other, which might lead to the observed lack of difference between the entropies estimated.

Additionally, we note that the distributions in Figure 10 are markedly different from the corresponding distributions presented in Figure 6. One can observe an evident underestimation of the presence of flat patterns, i.e., ‘0V’ in the case of deterministic pattern representation and ‘—P’ in the case of the dynamical pattern representation. In general, an overestimation of ‘1V’ (deterministic) and ‘↗P’ or ‘↘P’ (dynamical case) is present when the min-max normalization is used. The observed difference results from the substantial distinction between the two binning processes. Let us explain this using the following example: let a signal after the min-max normalization provide the bin size of 4 mmHg (compare with Figure 3 where the bin size is 3.7 mmHg) with bin edges 88,92,96,100,104,108,112. Then the segment values (98,101,100) are mapped onto (3,4,4), which is classified as a segment of ‘1V’/‘↗P’ in the deterministic/dynamical representation, respectively. In the case of the binning with bin = 4 mmHg applied to the same signal but when the bin edges are designed for each segment separately, the segment (98,101,100) provides bin edges 98,102,106,…, which leads to the flat pattern in any segment representation. Although the advantages and disadvantages of both the binning procedures could be disputed, the second one, using the segment-oriented bin edges, appears better to code the concept of pattern representation, namely it appears better to separate events greater than a given bin size, and for this reason improves the interpretation of the estimated quantities.

We counted how often values that differ less than a given bin size change the pattern classification of a segment. We found that, in the case of the adaptive, patient-oriented binning in both signals: RR-intervals and SBP value, independently of the time window, this occurs in, on average, 1/3 of the segments. On average, 12% of segments in T3 and 15% in H0 contain subsequent values that differ in less than half of the bin (classified as variate). Therefore, the estimates based on the signals binned with constant bin edges carry a large systematic error. In the case of segment-oriented binning, this kind of the systematic error only corresponds with the identification of ‘ΛP’ patterns. Fortunately, these patterns occur at negligible rates in the case of bins of size 3, 4 or 5 mmHg.

### 3.3. Couplings between Cardiac and Vascular Systems by TE

The 1969 concept of Granger causality [32] is straightforward: *Y* causally influences *X* if the knowledge of *Y* allows a better (more precise) prediction about *X*. Therefore, ${\mathrm{TE}}_{Y\to X}$ that quantifies the difference between predictions based on *X* and those based on *X* and *Y* is said to measure the information flowing from *Y* series to *X* series. In contrast to Granger causality [32], TE is capable of detecting nonlinear couplings. In the case of Gaussian variables, the two approaches have been proven to be equivalent [33]. Accordingly, TE has become an appropriate tool for investigations into the influence of the cardiac function on the vascular system and vice versa [34,35]. In particular, it is a potentially suitable measure for answering the question whether either of the two systems: cardiac or vascular, plays a leading role in preserving blood homeostasis after orthostatic stress. It might also be possible using TE to evaluate the dynamic exchange of the leading role between the two systems involved during the progression of orthostatic stress. In addition, of course, the possible balance scenario of equivalent influence of the two systems on each other in maintaining the blood distribution could be revealed.

However, similarly to ShE, the formulation of TE is based on the distributions of events. Therefore, before drawing any conclusions, one should test how the method of estimation of TE is specific to the signals examined, especially when the signals are represented by symbolized series. Let us, therefore, begin with the verification of the TE method by testing the influence of the past, which is taken into account in TE estimates. We then compare the results obtained for the pairs of the real data with the pairs of independent signals to filter out random effects. Finally, we discuss the message provided by TE group averages obtained in the subsequent time windows.

#### 3.3.1. Memory Length Influence on TE

**Figure 11:**
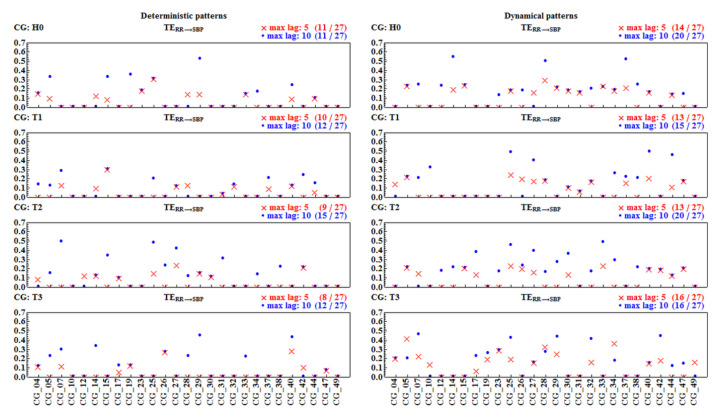
Transfer entropy ${\mathrm{TE}}_{\mathrm{RR}\to \mathrm{SBP}}$ from signals of each of the 27 subjects considered. Estimates were performed with memory vectors of length 5 (red crosses) and of length 10 (blue dots). In the upper right corners, the numbers of non-zero TE values are shown in the given time window and for a given length (max lag) of the memory vector.

**Figure 12:**
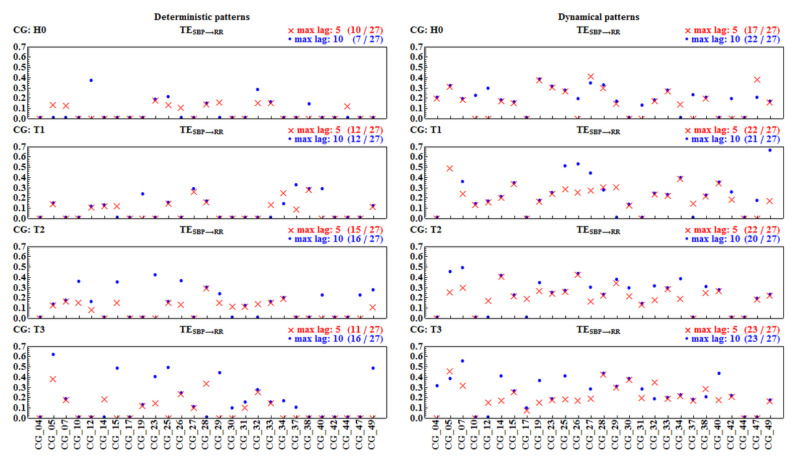
Transfer entropy ${\mathrm{TE}}_{\mathrm{SBP}\to \mathrm{RR}}$ from signals of each of the 27 subjects considered. Estimates were performed with memory vectors of length 5 (red crosses) and of length 10 (blue dots). In the upper right corners, the numbers of non-zero TE values are shown in the given time window and for a given length (max lag) of the memory vector.

Figure 11 and Figure 12 show values of ${\mathrm{TE}}_{\mathrm{RR}\to \mathrm{SBP}}$ and ${\mathrm{TE}}_{\mathrm{SBP}\to \mathrm{RR}}$ for each individual subject in all the time windows for the deterministic and the dynamical pattern representations. The results are obtained from the calculations performed with two lengths of the memory vector, L=5 and L=10.

The first surprising observation is that a substantial number of pairs of signals result in a TE of zero value. According to the numerical method used for the TE estimates (which follows concepts of MuTE [31]), this means that these pairs do not provide satisfactory statistics for the hypothesis that TE reveals information flow from one signal to the other. The next unexpected observation is that, although the doubled length of the memory vector results in an overall decrease in non-zero results, there were patients for whom TE became zero after the elongation of the memory. In most cases, the value of TE increased for L=10, but this was not the rule. Hence, manipulations in the length of the memory vector led to rather misleading results. Consequently, we decided to limit our further results to TE values driven by the last five beats, L=5, because the short-term dependence is the main objective of our investigations.

The descriptive group statistics (group TE mean ± StdErr, cardinality of the group and *p*-value of normality test) of the positive values of TE estimated with the memory length L=5 is shown in Table 3. We see from the table that the estimates for the information flow in both directions are similar to each other, although a slightly stronger role of SBP values on RR intervals in all time windows is observed when the signals are represented by the dynamical patterns. In the case of the deterministic representation, this prevalence is evident in the T1 and T3 windows only.

#### 3.3.2. Surrogate Signals’ Tests

**Figure 13:**
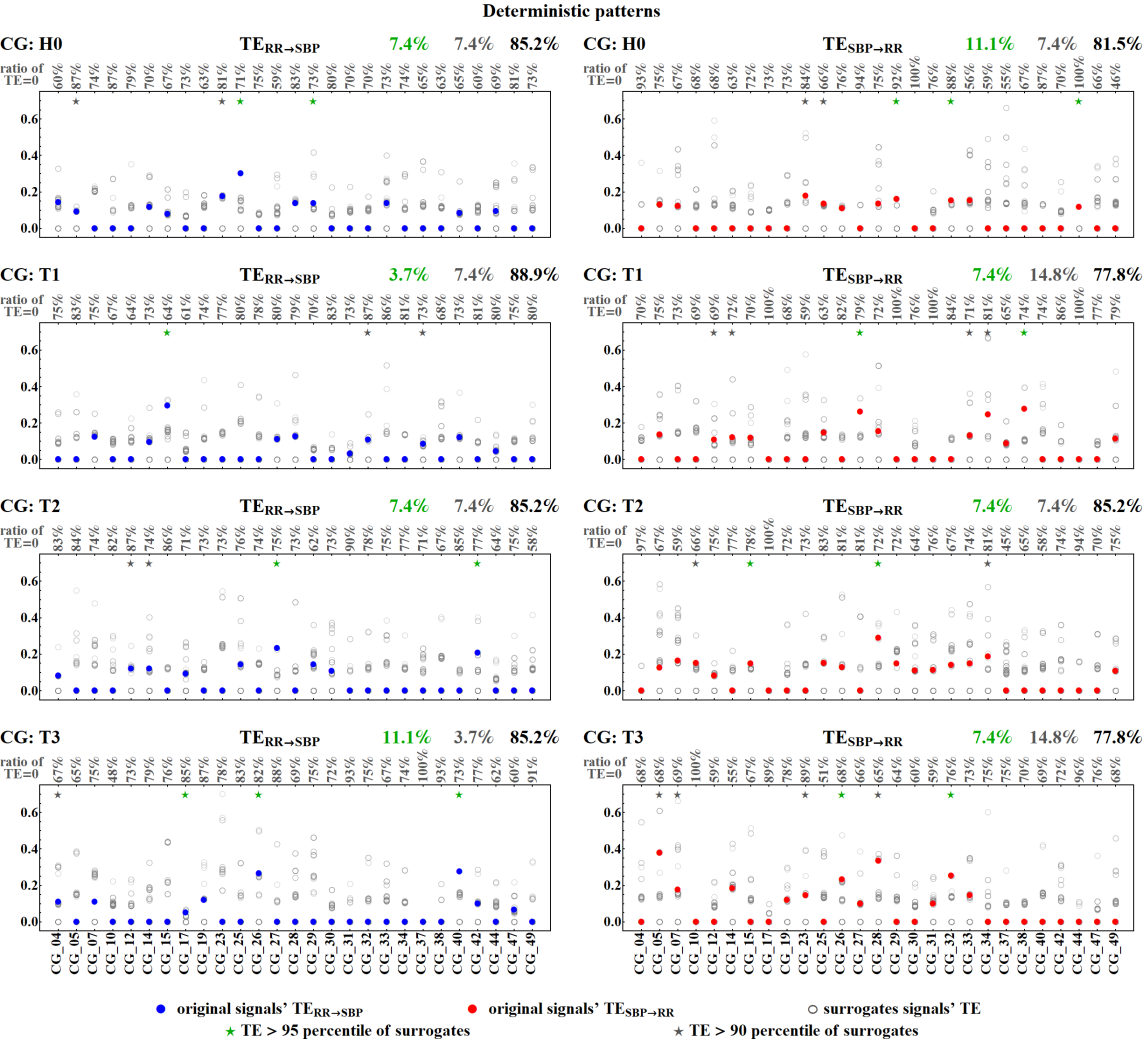
Statistics of transfer entropy ${\mathrm{TE}}_{\mathrm{SBP}\to \mathrm{RR}}$ and ${\mathrm{TE}}_{\mathrm{RR}\to \mathrm{SBP}}$ estimated from a hundred surrogates (grey empty dots) for each subject’s signals. The signals are represented by the deterministic patterns. The original signals’ TE are marked by filled dots. Green/grey stars indicate patients for whom the TE of the original signals was higher than 95/90 percentile of surrogates, respectively, and the total ratio of such signals is displayed above the plot. For each patient, the ratio of TE equal to zero among his/her surrogates is given. Remember that TE = 0 means the estimates are statistically insignificant.

**Figure 14:**
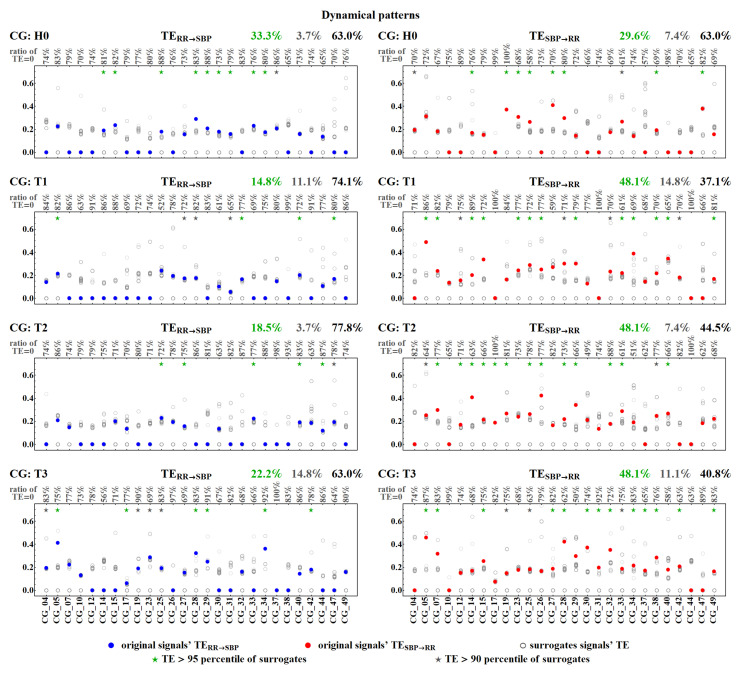
Statistics of transfer entropy ${\mathrm{TE}}_{\mathrm{SBP}\to \mathrm{RR}}$ and ${\mathrm{TE}}_{\mathrm{RR}\to \mathrm{SBP}}$ estimated from a hundred surrogates (grey empty dots) for each subject’s signals. The signals are represented by the dynamic patterns. The original signals’ TE are marked by filled dots. Green/grey 95/90 percentile of surrogates, respectively, and the total ratio of such signals is displayed above the plot. For each patient, the ratio of TE equal to zero among his/her surrogates is given. Remember that TE = 0 means the estimates are statistically insignificant.

Each signal with a pattern symbolization, by its construction, involves an intrinsic strong dependence between the last symbol and the two preceding symbols, which can prevail in TE estimates. Therefore, one should inquire whether the zero value of TE observed is due to the absence or weakness of the estimated couplings in a given patient, or whether it results from an insufficient sensitivity of the method applied in discerning the cardiovascular couplings. The test with surrogate signals enabled us to verify the influence of the direct interdependence between a given pattern and its last two preceding patterns in TE estimates. In Figure 13 and Figure 14, we present TE estimated from the two original signals but shifted randomly with respect to each other. For each patient, a number is displayed that provides the ratio of zeros occurring in the simulation experiments.

Among pairs of signals not adjusted in time, hence independent, approximately 70% led to zero TE. Hence, the estimates do not provide statistically significant values. In the set of the remaining non-zero results, one can investigate the relation between TE values obtained for the surrogate signals and the value of the TE provided by the unperturbed real signals. One can observe that only a few non-zero TE results survived random shifting, i.e., TE estimated for the original signals gave values statistically different (*p* < 0.05) from TE obtained for the shifted data. These statistically significant results are marked with green stars in Figure 13 and Figure 14. Their ratio with respect to the total number of signals considered is displayed in green above each plot. In general, these numbers are far from our expectations. Only in the case of the dynamic pattern representation, after the tilt, about half of the original signals provided non-zero ${\mathrm{TE}}_{\mathrm{SBP}\to \mathrm{RR}}$, in contrast to the surrogate signals. Thus, we can hypothesize that, after the tilt, dynamical patterns could be considered to be satisfactorily sensitive detectors of the factual influence of SBP values on RR-intervals.

#### 3.3.3. Couplings between Cardiac and Vascular Systems—A Patient Approach

To gain more insight into the relation between TE and an individual patient’s cardiovascular regulation, we propose considering TE as taking two values only: either non-zero ‘1’— there is an impact of one signal on the other, or zero ‘0’—the coupling is not present. This means that we ignore the strength of the cardiovascular coupling, but we concentrate on the timing, when TE detects the influence of one signal on the other or when it does not find such an influence. This approach allows us to observe concordance between values 0/1 discerned by ${\mathrm{TE}}_{\mathrm{SBP}\to \mathrm{RR}}$ and ${\mathrm{TE}}_{\mathrm{RR}\to \mathrm{SBP}}$ in the subsequent time windows for each patient individually. In particular, we focus on:

(a) any impact of the methods—deterministic or dynamical—of signal representation applied to see if the results of these methods are consistent with each other, or if they provide an independent description;
(b) the presence or absence of couplings in the different time windows: H0, T1, T2 and T3, to observe the development of couplings in the time windows;
(c) concordance of coupling directions SBP→RR and RR→SBP in the same window, to test whether the couplings between SBP values and RR-intervals instantaneously cooperate or rather follow each other.

To answer the above questions, we counted the coincidences of the events: (0 0), (0 1), (1 0) and (1 1) of TEs found for each individual patient with: (a) (different methods, same time window, same TE direction); (b) (same method, different time windows, same TE direction); (c) (same method, same time window, different TE directions). The differences in our counts from random tossing were tested by the McNemar test, p<0.05. It happened that only some of the proportions were significantly different from random occurrences. In particular, in the case of (a), satisfactory statistics were obtained for the transfer entropy of SBP→RR and only in time windows after the tilt. None of the (b) events reached satisfactory statistical significance, although in the case of the dynamical representation, ${\mathrm{TE}}_{\mathrm{SBP}\to \mathrm{RR}}$ between H0 and T2 and H0 and T3 was not far from being significant. Finally, referring to (c), the dynamical pattern representations after the tilt provided reliable relations between TE with different directions. Following the results of the statistical evaluation, we limit considerations only to the statistically meaningful variables.

Considering (a), we found that, for about 60% of patients, in the case of ${\mathrm{TE}}_{\mathrm{SBP}\to \mathrm{RR}}$ after the tilt, both pattern representations provide the same TE results. Hence, both methods consistently either do detect or fail to detect the SBP influence on RR. Among the remaining 40% of cases, it is interesting to note that the most pronounced difference between our counts and random event counts is the over-presence of (0,1) , i.e., events when ${\mathrm{TE}}_{\mathrm{SBP}\to \mathrm{RR}}=0$ if some signal is represented by deterministic patterns, and ${\mathrm{TE}}_{\mathrm{SBP}\to \mathrm{RR}}=1$ if that signal is represented by dynamical patterns. Together, an almost perfect absence is observed of the opposite event, namely (1,0). This observation might suggest that, in some subjects, there is a different organization of the response to the orthostatic provocation, which is not detectable by the approach of deterministic patterns. As dynamical patterns may be associated with baroreflex driven relations, and deterministic patterns can be seen as being especially sensitive to feedback corrections, we can further hypothesize that our results point to the prevalence of the baroreflex regulation. Thus, although each pre-processing method provides different results, together they lead to a more comprehensive description of the dynamics of cardiovascular regulation.

In reference to point (c), the method of dynamical pattern signal symbolization provides an unexpectedly high presence of pairs with zero values for ${\mathrm{TE}}_{\mathrm{SBP}\to \mathrm{RR}}$ (absence of vascular system impact on the cardiac system), accompanied by non-zero values of ${\mathrm{TE}}_{\mathrm{RR}\to \mathrm{SBP}}$ (presence of cardiac system impact on the vascular system) in the T1 time window. Then, in the T2 and T3 windows, these provide non-zero TEs in both directions. This fact points to a fast response of the heart rate to SBP, just after the transition in body position, which, however, does not produce an instantaneous reaction in the circulation system. However, then, with the passing of time, the vascular system affects the cardiac system. Therefore, the baroreflex loop, considered to consist of two parts: feed-forward (RR-interval to SBP) and feedback (SBP to RR-interval), can be seen as acting on two timescales. The first scale refers to the impact of the heart rate on the vascular system in a fast response to the change in body position, which then, later in time, evokes a vascular response.

## 4. Conclusions

Blood pressure is under permanent control served by a complex interacting network of neural and humoral systems. Together with local feedback loops, they distribute the blood all around the human body to meet the actual bodily demands. It is widely accepted that, as a result of the various cardiovascular regulation mechanisms involved, the variability emerges of both RR-intervals and SBP. However, the question might be posed as to whether this variability is an absolute, individual-independent property of the human organism, which, like body temperature, can be characterized by a single value defining a healthy state, or whether is it a relative, personal characteristic, which is attributed individually to each subject.

In order to shed some light on this problem, we considered signals that were first normalized and then binned. Therefore, one can say that the binning adaptively follows the individual signal values and provides patient-oriented, or patient-specific discertization. Next, the RR-intervals and SBP values were mapped according to two types of codes: deterministic patterns aimed at displaying the variability in a signal, and dynamical patterns, which, by revealing short trends focused on the overall system, aim to reveal the dynamics of speeding up or slowing down. Then, to reconstruct the supposed couplings involved in the cardiovascular system, two aspects of data exploration were addressed. Firstly, we investigated whether the entropic measures were robust and insensitive with respect to signal pre-processing. Secondly, by interpretation of the symbols used in the signal representation, we discussed the difference in the information about these couplings provided by short-term variability patterns versus short-term trend patterns. We have found that the complexity of patterns representing RR-intervals appears to be weakly dependent on the pre-processing method used, probably due to the strong non-stationarity during the abrupt transition dynamics of the HUT test. However, the complexity of patterns representing SBP values remains dependent on whether the signal was first normalized or not.

We have thoroughly discussed the possible reasons for the occurrence and the significance of the zero value of TE. We have found that limitations of the signal pre-processing and numerical methods applied appear to be the main reasons for the high percentage of TE zero values. Nevertheless, supported by statistical corroboration, we are able to discern meaningful dynamical aspects of the coupling between SBP values and RR-intervals, especially when the original signals are represented by dynamical patterns. In particular, we have found different timings evoking the cardiac impact on the vascular system from those evoking the vascular impact on the cardiac system—within the two basic mutually inter-coupled sub-components of the baroreflex loop.

## Figures and Tables

**Table 1:** Shannon entropy (ShE) from the distribution of patterns presented in Figure 6. In brackets, the entropy calculated from the corresponding shuffled signals is shown.

Patterns/Time Window	H0	T1	T2	T3
**RR-intervals**
ordinal	2.33 (2.55)	2.04 (2.56)	2.08 (2.56)	1.97 (2.56)
deterministic	1.27 (1.19)	1.16 (1.17)	1.21 (1.19)	1.16 (1.19)
dynamical	1.48 (1.55)	1.36 (1.53)	1.40 (1.54)	1.34 (1.54)
**Systolic Blood Pressure**
ordinal	1.93 (2.56)	1.92 (2.56)	2.04 (2.56)	2.03 (2.56)
deterministic	1.13 (1.18)	1.13 (1.18)	1.16 (1.18)	1.17 (1.17)
dynamical	1.37 (1.53)	1.34 (1.53)	1.37 (1.54)	1.36 (1.53)

**Table 2:** Mean ShE H ± StdErr from individual patient entropies. Below, *p*-value obtained from SW normality test of the grouped data.

Patterns/Time Window	H0	T1	T2	T3
**RR-intervals**
deterministic	1.21± 0.03	1.12± 0.03	1.17± 0.04	1.11± 0.04
*p*-value (SW test)	0.43	0.37	0.03	0.00
dynamical	1.43± 0.03	1.33± 0.03	1.36± 0.04	1.30± 0.04
*p*-value (SW test)	0.09	0.29	0.02	0.01
**Systolic Blood Pressure**
deterministic	1.05± 0.04	1.02± 0.08	1.09± 0.04	1.10± 0.06
*p*-value (SW test)	0.20	0.00	0.09	0.00
dynamical	1.30± 0.04	1.25± 0.08	1.31± 0.04	1.29± 0.07
*p*-value (SW test)	0.08	0.00	0.01	0.00

— deterministic: Kruskal–Wallis one-way ANOVA on ranks provided that RR-intervals gave statistically significant differences in medians (p<0.001); the pairwise comparisons (Tukey test) found group H0 different from both groups T1 and T3, SBP signals did not indicate statistically significant differences (p=0.12); — dynamical: one-way ANOVA indicated that RR-intervals provided statistically significant differences in means (p<0.001); the pairwise comparisons (Holm–Šidák test) found group H0 different from groups T1, T2 and T3, SBP signals did not indicate statistically significant differences (p=0.85).

**Table 3:** Mean transfer entropy ± StdErr estimated from pooled results obtained from signals of patients. Below *n* as cardinality of the non-zero TE group and *p*-value obtained from SW normality test of the grouped data.

Patterns/Time Window	H0	T1	T2	T3
${\mathrm{TE}}_{\mathrm{RR}\to \mathrm{SBP}}$
deterministic	0.14± 0.04	0.11± 0.06	0.14± 0.04	0.14± 0.07
*n*, *p*-value (SW-test)	11, 0.00	10, 0.01	9, 0.22	8, 0.03
dynamical	0.19 ± 0.02	0.16 ± 0.03	0.18 ± 0.02	0.21 ± 0.05
*n*, *p*-value (SW-test)	14, 0.52	13, 0.88	13, 0.35	16, 0.70
${\mathrm{TE}}_{\mathrm{SBP}\to \mathrm{RR}}$
deterministic	0.14 ± 0.02	0.16 ± 0.04	0.15 ± 0.03	0.20 ± 0.06
*n*, *p*-value (SW-test)	10, 0.89	12, 0.01	15, 0.00	11, 0.16
dynamical	0.24 ± 0.05	0.23 ± 0.04	0.24 ± 0.03	0.23 ± 0.04
*n*, *p*-value (SW-test)	17, 0.04	22, 0.19	22, 0.06	23, 0.02

Kruskal–Wallis one-way ANOVA did not provide statistical significance for differences in the medians for any group considered.

## Abbreviations

The following abbreviations are used in this manuscript:

ShE	Shannon entropy
TE	transfer entropy
RR-interval	time between two subsequent heart contractions
SBP	systolic blood pressure
HUT	head-up tilt
